# Report on Cerebro-Spinal Meningitis

**Published:** 1866-10

**Authors:** J. S. Jewell

**Affiliations:** Prof. Anatomy, Chicago Medical College; Member of Illinois State Medical Society, etc., etc.


ARTICLE XXXVIII.

### REPORT ON CEREBRO-SPINAL MENINGITIS.

By J. S. JEWELL, M.D., Prof. Anatomy, Chicago Medical College; Member of Illinois State Medical Society; etc., etc.

Read to the Illinois State Medical Society, June, 1866.

ACKNOWLEDGMENT.

To Drs. J. M. Woodworth and E. O. F. Roler, who have kindly exerted themselves, while engaged in foreign study and travel, to procure for me European monographs on the subject of this report; to my colleagues, Profs. Johnson and Byford; and to my friends, Dr. E. Marguerat and Mr. Eppler, of this city, my especial thanks are due, for valuable assistance rendered. The latter gentlemen translating for me a number of German monographs.

Within the past few years, a disease has made its appearance in various parts of the United States, pursuing, as a general thing, a rapid course, not very amenable to treatment, and attended by a fearful mortality; and in which the cause, whatsoever it may be, has seemed to expend its influence, mainly, on the contents of the cerebro-spinal cavity.

The disease was comparatively new to most of the practitioners of this country, and from this cause, and its widespread or epidemic prevalence and unusual mortality, it excited, very naturally, much attention among physicians, and alarm among the people. That the disease has frequently occurred both in Europe and other parts of the East, and in our own country in times past, will appear from a brief view of its history.

In giving an historical sketch of a disease about which so many opinions have been and even now are held, it will be necessary to keep in view, not only the chronological order of such occurrences of the disease as shall be given, but a more or less critical examination should be made of the reputed occurrences of the disease for reasons which will appear hereafter, and in which the attempt shall be made to eliminate all doubtful examples of its occurrence, that in this way an evident source of fallacy may be avoided. For the disease under consideration has often been confounded with other forms of disease distinct from it, and *vice versa.* But while an eye will be had to this matter, no formal attempt at a critical history of the disease will be made in this report.

In the historical sketch which follows, I shall first trace it in the Eastern Hemisphere, and, subsequently, in the United States. I will make no pretensions, here, to give all the occurrences of the disease that have been recorded, or that I have in my possession, but examples will be given from each well-marked epidemic. The sketch which follows, has been collected from a great variety of sources, both in this country and Europe, none, so far as I am aware, giving a consecutive history of the disease:—

HISTORY IN THE EASTERN HEMISPHERE.

From the best information I have obtained, it appears "cerebro-spinal meningitis" has prevailed epidemically in Europe and adjacent parts, chiefly in the south part of Europe, as follows:* In 1310, in France, which has seemed to be its favorite district in the Eastern Hemisphere. Then, in Europe again in 1503, 1510, 1516, 1517, and in 1528 once more, and was *this* time the precursor of the plague. In 1553, it appeared in Silesia, and carried off large numbers of the people. In 1556, it was observed in England and France; in Spain, in 1557, where it proved fearfully fatal; and in 1559, at Palermo, in Sicily. It occurred in many places in 1564. From 1569 to 1574, it prevailed in many parts of Europe, and was again followed by the plague. According to OZANAM, it appeared in 1580 at Rome, associated with a catarrhal affection, and swept away not less than 12,000 persons; and in like manner, 12,000 at Venice; and 2000 at Madrid, in Spain. In Europe again, in 1582. It was observed at Trent in 1591, and in 1592 at Florence. In various parts of Europe, in 1616 and 1624. It is reported by SYDENHAM, in 1661, as partaking of the charac-

* I have not been able, as yet, to obtain the report of Dr. BURDON SANDERSON, made to the British Parliament, on the disease as it appeared on the Lower Vistula; nor the elaborate monograph of MANKOPFF'S, which has been, or is to be, published at Vienna, in Austria, but *will* soon as possible.

ter of typhus. In Italy, in 1691 and 1693, and in England, in 1698. In Prussia, in 1704, and again in England, in 1710 and 1741. In Piedmont, in 1720, in Egypt, in 1760, and again in Europe, in 1761. According to MARTEAU DE GRANDVILLIERS, at D'Aumale, in 1757 and in 1778, coincident with an epidemic of typhus at Lyle; and again, in 1778, in other parts of Europe. In 1805, it appeared at Geneva; then at Melazzo, in Sicily, in 1808, where, according to one BOYLE, the most common characteristic seems to have been plastic effusion within the cerebro-spinal cavity. In 1814, it appeared at Grenoble, and was reported on by Mons. BILLERY, and after this occurred at Vesaul, in 1832.

In 1837, it appeared at the military hospital at Bayonne, where 33 persons soon fell victims to the disease. From this date, the disease spread northward through France, in two directions, and, during the time of its progress, no less than 57 epidemics are reported, many of them attended by an alarming fatality. Of the two paths of progress of the French epidemics, one extended along the eastern, and the other along the western border, penetrating but once into the interior of the country.

The western path began at Bayonne, as already related, and extended over Bordeaux to the Loire, visiting La Rochelle and the province of Landes, Dax, etc. It extended up the Loire, on either hand, into the interior, of which this was the only example. It then appeared at Brest, Cherbourg, Caen, and other places on or near the coast, in 1840 and 1841, and from these places it extended onward to Versailles, about Lothringen, Elass, and Languedoc. Between 1839 and 1842, it appeared at Metz, Strasbourg, Nancy, Colmar, and, still farther, at Laval, Chateau Gonthier, Tours, Blois, Joigny, and in the neighborhood of Rambouillet.

Following the other branch, which began at Narbonne and Foix, and dates at 1837:—In 1838, it appeared at Toulon, and early in 1839, at Nismes, and in the winter of this and the following year at Avignon. Here, it was mainly confined to the military, as in some other parts of France, though it appeared many times among the civil population. It was then seen at Mont Brison, in 1840 and 1841. The winter following, at Aigues Morte, and, occasionally, elsewhere, until 1849.

In Italy, the disease appeared in 1837, at Terra Di Savolo. It then extended to Calabria Ulteriore, and Principato Ulteriore, and also was observed at Naples, in which latter place it again appeared in 1846, and in various parts of Sicily. Since then, the disease has, until lately, not prevailed in Italy. In the winter of 1865, it reäppeared at Nizza and vicinity.

It was observed in Algiers from 1839 to 1847, especially the eastern part, and from here it extended toward the great Sahara desert.

Besides the places already mentioned, it appeared at Gibraltar in 1844, mainly confined to the native population.

From 1845 to 1848, it prevailed with great malignancy in Denmark. In 1846 and 1847, it was observed in Ireland, especially in the vicinity of Belfast, as reported by Dr. R. Mayne, and also at Edinburgh, in Scotland. From 1854 to 1861, it prevailed in a large number of places in Sweden, also at a few places in Norway, and, about the same time, among the troops from Holland, at Anheim. The disease was observed by Reinacher, at Wrisburg, in 1851. In 1863-4-5, it appeared in many parts of Germany, but especially the Dantzig district of West Prussia, as related by Hirsh, Rummel, and others, where, perhaps, 2000 cases occurred; and, also, less extensively, in other parts of West Prussia, as at Berlin, Bromberg, Stettin, etc.; and farther east, in the Grand Duchy of Baden, at Carlsruhe, and Rastatt, as related by Niemeyer, of Tubigen.

HISTORY IN THE UNITED STATES.

Let us now turn to its history within the limits of the United States. Though I have somewhere seen a statement of its earlier occurrence, yet the earliest recorded occurrence of the disease in this country which I now have at hand, is that at Medfield, in Mass., in 1806, and, again, in Litchfield County, Conn., in April, 1807. From this time, during the years of 1807-8-9-10, it was observed, winter and spring, at many places in the New England States and New York, and by 1810-11 the disease had begun to prevail extensively and with great severity, in New England, New York, and Canada. In New England it reached, perhaps, its highest degree of prevalence during the year last mentioned. From this period the disease began to decline, and, finally, in the course of a few years disappeared as an epidemic. This epidemic was reported on by many observers, among whom were GALLUP, NORTH, WARREN, WELCH, STUART, THACHER, HOSACK, MINER, and TULLY, etc. It was coincident with a marked typhus constitution, and cases were frequently complicated with pneumonia and pleuro-pneumonia.

Between 1840 and 1850, it prevailed epidemically in many parts of the Southern, Middle, and Western States, as in Alabama, Tennessee, Kentucky, Missouri, Illinois, Indiana, and Michigan. Since that time, the disease has not appeared epidemically, so far as I can learn, or, at least, notably so, until since the beginning of the late war. From the winter and spring of 1862-3, the disease has prevailed in a large number of localities, both North and South, and many times, but especially in the Southern States, attended by a fearful mortality. Happily, at this time, the malady has subsided as an epidemic, though sporadic cases are not unfrequently met with.

Accounts by various observers have been given of the late epidemics in this country and Europe, but few of them, however, for various reasons, satisfactory as desirable; and to these reports, and to such accounts of earlier epidemics as I have been able to possess myself of, or the *facts* they give us, let us now turn our attention.

### MANNER OF TREATING THE SUBJECT.

Before I go farther, it may be well for me to say something about the method I have adopted in treating this subject. I have obtained many of the most important reports, both in Europe and this country, and where I have been unable to consult a report directly, I have frequently done so through friends who had access to them. The matter I have thus become possessed of has been analyzed, and all facts of the same kind or order arranged under appropriate heads. Four tabular statements of facts were made in this way; the first, exhibiting the symptomatical; the second, the *post mortem;* the third, the etiological and pathological; the fourth, the therapeutical histories of the disease. From these analyzed statements, the body of the report has been drawn. Following this, is a discussion of the facts, in which the attempt is made to evolve their teachings. In each statement, room was left for incidental facts and exceptional cases.

In the examination I have made into the literature of this subject, I have remarked such a contrariety of opinion, as to its nature, etc., in cases where an opinion has been offered at all, and such a difference in the appearance of the disease in some particulars, as it is described by various observers, as to impress me with the belief it was necessary to make a broader survey of the disease, as it has appeared at various times and places, and, in this way, to collect a greater number and variety of facts than has been customary with those who have considered this subject; that in this way an evident source of fallacy may be avoided, and a broader foundation laid for sound deductions.

In an inductive science like medicine, *facts* lie at the foundation of true advancement, and in proportion as they are numerous, in the same degree are we likely, all else equal, to reach truthful conclusions. For the purpose of obtaining such a body of facts as might lead to sound conclusions, I have taken some pains to collect them; and in going to all available sources, I have done so, actuated only by a feeling of the above-mentioned necessity.

And surely, a disease which has occurred so often, and concerning which there is such a body of material within reach of the profession, but of which the profession, as a body, knows so little, and which has been so imperfectly understood, has proved so intractable to treatment, and which involves so many interesting questions in pathology. I say, surely such a disease demands a careful study. For these and other reasons, I have felt compelled to extend this report beyond what I had originally intended as it limits.

### I. SYMPTOMATICAL HISTORY.

*1. Premo itory Symptoms.*—It is pretty well agreed among observers that there are no well-marked premonitory symptoms which may be regarded as characteristic of the disease. Many times, from a state of apparently perfect health, the patient is seized with the disease, and without warning. *E.G.*, M. TOURDES saw premonitory symptoms in 44 cases out of 73. HIRSH relates, that out of 40 cases, 24 had similar premonitory symptoms, 16, however, had none, and some were, as stated above, attacked *ab initio*, with the fully developed disease, even while at their ordinary avocations.

But, generally, the attack is sudden. The most constant of the *early* symptoms is that of *chilliness*, which sometimes amounts to mere rigors, but more often to a severe chill. NIEMEYER has seen cases occur without a perceptible chill, but such cases must be very rare.

Another symptom, almost constantly present, is *pain in the head*, extending frequently down the back of the neck, or even down the full length of the spinal column. HIRSH noticed pain in the abdominal walls, as one among the earliest symptoms. Drs. SKIVEN, GREENE, and others, have noticed pain in the knee, or some part of the extremities, before pain in the head; but in almost every case it has soon appeared in the head, and from thence has frequently extended to the back and extremities, especially the lower. There is sometimes, quite early, soreness in the back of the neck and elsewhere.

*Nausea* and *vomiting* have also been observed as among the earliest symptoms, as well as most common. The bowels have generally been *normal* or *constipated*. Some *few* observers, however, have reported severe purging, like in cholera or cholera-morbus. But my enquiries show any form of looseness of the bowels to be rare.

Sometimes there is a sense of stiffness and soreness about the throat, as recorded by GALLUP, and stiffness of the muscles of the face and neck, and sometimes slight paralysis of the tongue was observed by Dr. UPHAM.

*Delirium*, although not so early as the symptoms already referred to, is, yet, very soon, one of the most constant and characteristic symptoms of the disease, and, in a few cases, is soon followed by coma, or even may be preceded by *coma.* Dr. ARMSTRONG, of Mobile, saw the delirium commence with extreme suddenness and violence.

The *pulse* is, *generally speaking*, diminished in force and volume, and increased in frequency. The *surface* is usually paler than natural. The condition of the *pupils* not changed or variable. Such are the most prominent of the early symptoms. Others have been reported, but they are not sufficiently constant to deserve attention.

All the premonitory symptoms indicate a most serious involvement of the nervous system, especially the cerebro-spinal, and point, unmistakably, to those parts as the seat of the disease in which, as we shall see, *post mortem* examinations reveal the greatest changes.

*2. Surface.*—In the beginning of almost every case, the *temperature* of the surface is lower than natural, and in some virulent cases remains so till death. After reaction occurs, the heat of the body generally does not rise much above what is natural, though, in a few cases, it becomes excessive. In the cases observed by HIRSH, the temperature was not, on an average, perceptibly increased above natural, as indicated by the thermometer in the axilla.

The *moisture* of the skin has been variable. Sometimes it is dry as well as cool, as observed by ALLEN and others. 25 per cent of the cases observed by the gentleman last named, are said to have presented a moist skin. According to RUMMEL, after the disease had progressed a few days, occasionally profuse perspiration occurred at irregular intervals, sometimes only partial, as on the head and face, at other times over the whole body.

The *color* of the skin has been variable, perhaps, most frequently nothing remarkable. In the beginning of the disease, it is generally paler than natural; especially is this true of the face. The surface has sometimes been observed of a cyanotic hue, as by HIRSH and OSTER. Sometimes the face is flushed after reaction, as in the cases reported by UPHAM and others; rarely so from the beginning. It is owing to the fact that *maculæ* or *spots* of various kinds appear on the skin occasionally in this disease, that it has its name "spotted fever." Spots of various kinds have been observed in this disease, chiefly as follows:—

*Petechia* have been most frequently observed. They vary much as to time of appearance, extent, color, and duration. They seldom appear at the commencement of the disease—frequently about the second or third day or later—while, in the majority of cases, they never occur with any distinctness at all. They seldom are copious, and generally are confined to the neck, breast, and arms, appearing frequently on other parts of the body. As to color, they vary from red to a purple or dark color, and may appear at almost any period, and disappear at any time after their occurrence. They have sometimes been observed to persist until after death. They vary in size from a pin's head to half-an-inch or more in diameter. They are generally distinct, though they may be confluent. They are seldom elevated, and do not usually disappear on pressure. Most observers agree they are not pathognomic, and that their appearance or disappearance has no marked import, either in diagnosis or prognosis. Some observers who have met the disease have not seen them at all. NIEMEYER observed them occasionally in the epidemics at Carlsruhe and Rastatt. RUMMEL and HIRSH saw them very seldom in the Dantzig epidemic. They were observed by FORGET at Val de Grace but seldom. BROUSSAIS and LEVY mention them in a few cases. They were seen more frequently by BOUDIN, MATTOT, TOURDES, and LEFEVRE, and among the older writers, they are mentioned by PRINGLE, HILDENBRAND, and others.

In the epidemic which prevailed in the New England States, between the years of 1808 and 1812, they were observed by Dr. GALLUP, about one time in six, distinctly. They are mentioned as frequently seen by Dr. NORTH, and those to whom he refers, in his work on "Spotted Fever." In our own late epidemic, they were more frequently seen than in the contemporaneous epidemics in Europe. They are mentioned by numerous observers, as Greene, Gerhard, Draper, Upham, Levick, Allen, and others, who have recently communicated their observations to the medical journals of the country.

As regards the character of the spots, they seem, generally speaking, to be extravasations beneath the skin, and are seldom elevated to the same degree they often are in typhus. They seem *sometimes* to be simply congested spots, which more or less disappear on pressure—the former do not disappear thus.

*Herpes Labialis.*—After the spots referred to in the last paragraph, herpes has been most common. Most observers in Europe speak of it. It has been noticed by Forget in a large number of the cases which came under his observation. The same happened in Niemeyer's cases. Hirsh and Rummel saw herpes frequently in the Dantzig epidemic; also M. Levy, at Val de Grace. The eruption has generally appeared about the mouth, and on various parts of the face, extending from thence to the ears, neck, etc., rarely farther. It appears at irregular times, and seldom in the rapidly fatal cases. It generally occurs in those cases which have several days' duration and are distinctly febrile in character.

Occasionally other eruptions, as roseola, occur. Oster and Hirsh have seen this. The latter has seen a rash like that of scarlatina, or like measles, in a very few cases. While Levy and others have seen erysipelas coincident with the disease.

Such are the most important facts relating to the surface of the body which have been observed. It is to be remarked that none of these appearances are pathognomic, since, in the majority of cases they do not appear at all, or, at least, distinctly, and since many diseases, in many respects different from this one, present the same phenomena.

*3. Vascular System.*—The character of the pulse, generally speaking, from the beginning to the end, as noted by a large majority of observers, has been:—1. That of diminished force. 2. That of increased frequency, and, 3. Less frequently irregularities. By Niemeyer, the pulse was found to correspond to the temperature. The pulse in cases where the temperature was low, was observed, in a few cases, as low as 60 in a minute. The pulse was noticed by HIRSH, RUMMEL, and NIEMEYER as generally slow at the beginning of the disease, but more frequent as it progressed, especially in fatal cases.* Such was the experience of GALLUP and others in our own country, and of the French observers. This feebleness seems to be shared by the entire circulatory system, heart and bloodvessels alike. But exceptions occur. In one case, which proved fatal three hours from the date of attack, the pulse was normal in frequency, and was full, and so continued until after respiration ceased. This occurred in my own practice. Such cases, however, are very rare. *Hemorrhages* have seldom occurred. *Epistaxis* has, perhaps, been most frequently observed. LEVY, *e.g.*, says that out of 43 cases, 9 had epistaxis. Hemorrhagic spots also occur beneath the skin and mucous membranes, and sometimes beneath the serous membranes and into the texture of various organs, as will be shown more fully in the *post mortem* history of the disease.

*4. Blood During Life.*—It is a matter for regret, that the blood has not more frequently been examined. But in all the cases which have fallen under my notice, the fibrine has uniformly been increased in quantity, or above the normal standard, and the same remark has been true of the corpuscles. The color of the blood has almost uniformly been bright. In 4 cases, in which the blood was examined by ANDRAL and GAVARRET, the fibrine was found increased from 3.70 to 5.63, the normal standard being about 2.20; and the corpuscles from 134 to 143 parts in 1000, the normal standard being about 131. Dr. AMES, of Alabama, examined the blood in 4 cases, and found the fibrine from 3.46 to 6.40 in 1000. Mons. MAILLOT reports 6 cases, where the blood was examined by SOULIER,

* I shall make more frequent reference to some foreign monographs, than to the brief papers published in our own country, *only* because some of the former embody such exceeding careful, extended, and valuable observations. I am deeply indebted to the excellent monographs of RUMMEL, HIRSH, NIEMEYER, and KÖHLER. The latter is one of the most elaborate treatises ever published on all forms of cerebro-spinal inflammation. Their titles, with others, will be found elsewhere.

where the fibrine rose to 6 parts in 1000. I have, myself, had the opportunity to observe the same fact. In one case, blood was drawn the third day of the disease, it was the first bleeding. It coagulated rapidly, and with a clot which, for size, firmness, and thickness of the buffy coat, surpassed anything I have ever seen. The objection which has been made to such examples, that the blood was probably examined after the second or third bleedings, when we would be led to expect an increase of fibrine if it were true, as it is not, will, certainly, not apply to the corpuscles, since we should, for the same reason, look for a diminution of the corpuscles. But we have seen they are increased. Observations, tending to establish the same facts, have been made by M. FORGET, FAURE VILLAURE, and others. The buffy coat has been noted as often very considerable by numerous observers. Dr. AMES abstracted blood in 37 cases, and in all the color was unusually bright and the coagulation rapid. In 4 cases out of 37, he witnessed the buffy coat.

5. *Digestive System.*—The *tongue* is frequently normal, as mentioned by BROUSSAIS, *e.g.* Usually it has a whitish coat, sometimes creamy, and is moist. There are, however, numerous exceptions to this statement. After the disease has progressed for a time, and, rarely from the beginning, the tongue is yellowish or brownish, and, *sometimes*, dry. But in the latter stages of most cases, especially fatal cases, the tongue becomes brown, or even dark, and fissured and dry, and not unfrequently, in such cases, red, and, in a few examples, sordes collect on the teeth, lips, and tongue. BROUSSAIS reports the tongue brown or dark from the beginning, rarely. ALLEN has seen the tongue swollen, and presenting indentation along the margin.

The *stomach.* The most constant symptom referable to the stomach, is *nausea* or *vomiting.* Indeed, so early and so constant is this symptom, that it is one of the most characteristic of the disease. It is increased by assuming the upright posture. It is almost exclusively sympathetic, and is usually referable not directly to the stomach, but to cerebral disorder, and is generally accompanied by fever and headache. It almost invariably occurs within the first two days of the disease. It generally passes away after a day or two, but may continue for several days, or recur at intervals. In a few cases, it has not been observed at all. There is, usually, nothing peculiar about the matters vomited, though bile has often been observed, as by Dr. ARMSTRONG, of Mobile. The appetite is variable, though generally impaired or lost. The bowels have generally been observed as constipated or normal. Diarrhœa seldom occurs, but has been observed, rarely, as severe.

*6. Respiratory System.*—But few symptoms occur here worthy of note. Most observers state, on the average the respirations are slower than natural in this disease, though there are marked exceptions. As the disease advances, especially in fatal cases, the respirations become more slow and laborious till death. They are also frequently irregular. At the commencement of the disease, and many times during its progress, the respirations become sighing and oppressed; or they may be increased in frequency, as was often observed by NIEMEYER and the French observers; but in uncomplicated cases they are normal, or slower than natural. It is scarcely worth while to mention that in a few cases bronchitis, or pneumonia, or simple engorgement of the lungs have occurred, indicated by the usual rational and physical symptoms and signs. But such complications have been, so far as I have examined, purely incidental.

*7. Secretions.*—It is matter for regret, that the state of the secretions, and especially that of the urine, has not more frequently been examined with care. The *quantity* of the urine, though somewhat diminished at various stages of the disease, was usually not far from normal. It has been seen increased in quantity, and of low specific gravity, by ALLEN, while on the contrary, HIRSH has seen it frequently, at the beginning, of of high specific gravity. Some few observers report it generally scanty.

*Quality*, so far as known, not far from normal. Dr. ROOKER, of Indiana, mentions a high color and excess of the urates, sometimes. HIRSH mentions one case, in which he saw a precipitate of the triple phosphates, and another, complicated with typhus, in which the urine was deeply colored, was highly acid, and afforded copious turbid deposits of the urates. ALLEN generally found the urine smoky, albuminous, depositing sediment, and containing disorganized blood corpuscles. The urine of patients convalescing slowly was found, invariably, albuminous. Drs. LODGE and SAMUELS, of Marion, Illinois, report the urine sometimes albuminous.

The *perspiration* presents nothing peculiar. Some observers have seen copious perspiration at irregular periods of the disease, either general or partial. I have seen it most copious in those cases where the temperature of the surface was highest. Profuse perspiration has been, comparatively, oftenest observed by GALLUP, WALES, and ROOKER. The first two observers report a mawkish odor arising from the skin during perspiration.

*8. Nervous System.*—The symptoms referable to the nervous system are, almost beyond measure, the most conspicuous and important, and demand a large share of our attention. Whatever other symptoms are absent, referable to other organs, or systems of the body, symptoms which refer to the nervous system are never absent. As a general thing, all the functions of the nervous system give evidence of derangement at some period in the course of the disease. We shall range these symptoms under three heads:—1st. Motor. 2d. Sensory. 3d. Psychical.

1st. *Motor Disturbance.*—The state of the voluntary muscular system indicates the condition of the motor part of the cerebro-spinal nervous system. Two sets of phenomena appear: (*a.*) Involuntary muscular action in various degrees. (*b.*) Paralysis of motion. The former is by far the more frequent, and gives us, tonic spasm; clonic, or epileptiform spasm; and, finally, jactitation, or restlessness.

(*a.*) 1. *Tetanic, or Tonic Spasm.*—This is almost a constant symptom of the disease, and varies greatly, as to its extent and time of appearance. Rarely, cases have been seen without this symptom in any noticeable degree. It sometimes appears at the commencement of the disease; but commonly does not appear in any marked degree until after the onset. It is usually preceded by a sense of stiffness, occasionally of soreness, in the affected muscles, far more frequently the former. HIRSH noticed painful twitchings to precede the rigid state of the muscles. It is also one of the most persistent symptoms, continuing many times far into convalescence. In a few cases it may disappear early, before death, or before recovery, but generally continues until death, or slowly disappears on recovery. Its appearance may be either gradual or sudden. The muscles almost invariably affected, are those of the back of the neck, and, next in order, the extensors of the back and flexors of the limbs. Cases have occurred, in which the muscles of the back have been alone affected, but they are rare. Trismus is, perhaps, next in order of occurrence. Cases presenting this symptom have generally proved fatal. Next in order, strabismus, perhaps, sometimes both eyes, generally one, and that one usually the left, according to HIRSH. It is almost without exception converging. Pleurosthotonos has been observed, as by M. LEVY, at Val de Grace. Contraction of the abdominal muscles, and of the flexors of one or more of the limbs, has been observed. Perhaps not anywhere, more appropriately than here, can we state the condition of the pupil. It is variable. It may remain normal, as has been observed by FORGET and others, and may be either contracted or dilated throughout the disease. But, generally speaking, the pupils are contracted in the early part, and dilated in the latter part, especially in grave or fatal cases. Occasionally, one pupil has been observed contracted, while the other has been dilated. [NIEMEYER.] In delirium, they are generally contracted or *variable*, and in some degree sensitive to light. In coma, they vary, but most times are dilated, and usually insensible to light. In rapidly fatal cases, in which coma appears early, and also paralysis, the pupils are generally dilated the whole course of the disease.

2. *Clonic, or Epileptiform Spasm.*—Convulsions are far less constant than tetanic rigidity of the muscles. We may have them of all degrees of severity, from simple tremors or twitchings of the extremities or muscles of the face, as recorded by HIRSH and others, to severe general convulsions. They may be partial, as of one arm, or leg, or finger, or of the face.

They may occur at any period of the disease, but are not seen in any marked degree in the majority of cases, perhaps. They are more frequent in young than adult persons.

Except in cases comatose from the beginning, or in which the coma is profound, there is generally restlessness. This must not be confounded with the tremors, etc., already mentioned. The latter are true convulsive movements, and may and do take place when the patient is wholly unconscious. The restlessness is due to the severe pain which is felt in a half-conscious state, just sufficient to arouse the patient to movements expressive of distress.

(*b.*) *Paralysis of Motion* is, comparatively, not frequent. It has been observed of various degrees, and at various periods of the disease. It is rarely general. Such cases are usually fatal. Partial paralysis is far more common. Hemiplegia and paraplegia have been observed. Paralysis of one limb has been noted, and paralysis of the muscles about the face often occur. Sometimes dysphagia, or loss of articulating power have been noticed. HIRSH and NIEMEYER have recorded cases of acinesia, paresis, ptosis, etc. Such cases as present the last two symptoms generally proved fatal.

Paralysis seldom appears at the beginning of the disease—usually after it has advanced somewhat—most commonly in the later stages. It may disappear suddenly, or persist until death, or slowly disappear with recovery, or, finally, it may prove persistent (though rarely) after recovery. This class of symptoms are, as a general thing, more unfavorable than the preceding.

2d. *Sensory Phenomena.*—For convenience, we may consider general and special sensation apart:—

1. *General Sensation Exalted—Hyperæsthesia.*—Most observers have met with cases in which hyperæsthesia was present, and some, as HIRSH, have met them frequently. I have seen two cases, in children, in which the hyperæsthesia was almost the only *well-marked* symptom. HIRSH has *always* found it when present, associated with *tonic contraction of the muscles.* It sometimes appears early and persists till late in the disease, or into convalescence. It commonly affects the whole of the surface of the body, or nearly so, but may be partial. The surface is sometimes so painfully sensitive as that the slightest movement of the person, or simply touching the surface, or even a current of air produces excruciating distress, the patient complaining bitterly. This contributes to the restlessness and moaning of delirium, or even in somnolence or coma. It appears in some degree, either partial or general, at some period of the disease in, perhaps, the majority of cases.

2. *General Sensation Depressed—Anæsthesia.*—This is far more rare than hyperæsthesia. Like the latter, it may be either general or partial, and may disappear before death or recovery, but is seldom persistent. It usually occurs in the middle and later stages of the disease. In the cases observed by NIEMEYER, the mind was clear.

3. *General Sensation Perverted—Pain.*—Pain is one of the earliest and most common symptoms. Its most constant seat is the head. While it may be felt in any part of the head, its usual seat is the back part. It may be here, as elsewhere, various in character, but is generally severe, and is sometimes described as *terrible.* It is sometimes shifting in character. It is also persistent, being commonly one of the last symptoms to disappear in convalescence.

It is next most frequently observed in the back of the neck, where it partakes of the character of the pain in the head, but is not so constant. Next in order, is pain in the lumbar region, and after this, in the dorsal region and in the extremities. Pain is frequently referred to the peripheral extremity of a nerve, as to a limb, the face, etc. The only constant seat of pain is the head, though there have been exceptions. Pain in the abdominal walls was frequently and early observed by HIRSH. The pain was, as it generally is, increased by motion.

3d. *Special Senses.*—They are disturbed in a variety of ways and degrees. Impairment, and even loss of vision or hearing has not been uncommon. These disturbances might occur at any period of the disease, but generally in the middle and later stages, and in a few cases have proved persistent; same may be said of the other special senses. An exploration, by means of the ophthalmoscope, of the interior of the eye, determining in this way the degree of vascularity of the choroid, would seem, in this disease, to throw some light on the state of the brain or its circulation.

4th. *Psychical Phenomena.*—1. Under this head, the symptom which is most common, is *delirium*, observed at various periods and in different degrees. Cases have occurred, in which no prominent symptom of mental disturbance has appeared during the whole course of the disease, but they are rare. Such are the purely spinal cases, in which the contents of the cranial cavity are slightly or not at all involved. While it may appear either at the outset, or in the middle or later stages, it usually appears in the early part of the disease. It is usually remittent, and may be intermittent, or may alternate with coma. [FORGET.] It may come on suddenly or gradually. It usually does not appear until after the chill, headache, etc. It is not so constant as these symptoms, though some deem it characteristic. It may, as already remarked, exist in various degrees, from simple mental wandering when undisturbed, to that the most violent. It may be pleasing or amatory, or such as leads the patient to be fierce and untractable. The delirium generally precedes the coma, but FORGET, *e.g.*, has seen it otherwise. When delirium is present, the pupils are seldom dilated, may be normal, but are generally contracted in some degree and variable, and, as already remarked, in some measure sensitive to light.

2. *Coma.*—This symptom is not so constant as delirium. Out of 46 cases, Mons. FORGET saw 8 cases where coma set in at once. It occurs with the least frequency in the beginning of the disease, most so in the later stages. It is a more unfavorable symptom than delirium, and usually, though not always, indicates effusion, serous or otherwise. Those cases in which decided coma occurs, comparatively seldom recover, whether it appears early or late. It may, like the delirium, prove remittent or intermittent, in the latter case alternating with delirium. During coma, the pupils may be normal, or even contracted somewhat, though rarely. They are almost uniformly in some degree dilated and insensible to light. Coma may vary in degree, from simple dulness or somnolence to that the most profound.

*9. Periodicity and Stages.*—This disease is not characterized by well-marked stages. The symptoms are generally somewhat periodical or rythmical in their progress. This has been particularly noticed in localities where periodical fevers prevail. To such an extent has this been the case in some places, especially in the West, as to induce the belief among some physicians that it was a form of "pernicious" malarial fever. In such instances of the disease is it that the quinia treatment has proved so beneficial.

*10. Duration.*—The duration of the disease varies from a few hours, to weeks or even months. I have seen one case die within three hours from date of attack, and have seen another pass along for three months. RUMMEL remarked the disease to run its course sooner at the beginning of an epidemic, than at its close.

*11. Mortality.*—It is greatest during the first four days of the disease, and among the young; and at the beginning rather than at the close of the epidemic. HIRSH reports that of 391 fatal cases, 359 were under 15 years. NIEMEYER found that of 66 males, 20 died; of 60 females, 18 died; of 54 cases, from 1 to 5 years of age, 18 died; 40 cases, from 5 to 14 years, 9 died; 27 cases, from 15 to 23 years, 10 died; and in 5 cases, above 23 years, 1 died. The following mortality table of some of the epidemics in France, is mainly taken from the monograph of M. BROUSSAIS:—

| | | | | | | |
|---|---|---|---|---|---|---|
| Nancy, | 8 deaths in | 28 cases, | are | equal to | --------- | 1 in 3.50 |
| Le Mans, | 3 | " 9 | " | " | --------- | 1 in 3,00 |
| Ancenis, | 4 | " 12 | " | " | --------- | 1 in 3.00 |
| Mont Brison, | 16 | " 47 | " | " | --------- | 1 in 2.93 |
| Caen, | 4 | " 10 | " | " | --------- | 1 in 1.25 |
| Poitiers, | 8 | " 20 | " | " | --------- | 1 in 2.50 |
| Versailles, | 111 | " 227 | " | " | --------- | 1 in 2.04 |
| Metz, | 22 | " 40 | " | " | --------- | 1 in 1.81 |
| Perpignan, | 28 | " 50 | " | " | --------- | 1 in 1.78 |
| Strasbourg, (Mil. Hosp.) | 108 | " 184 | " | " | --------- | 1 in 1.70 |
| " (Inhab,) | 90 | " 150 | " | " | --------- | 1 in 1.66 |
| Laval, | 44 | " 69 | " | " | --------- | 1 in 1.56 |

| | | | | | | |
|---|---|---|---|---|---|---|
| Colmar, | 5 | " | 7 | " | " | ---------- 1 in 1.40 |
| Bayonne, | 21 | " | 28 | " | " | ---------- 1 in 1.33 |
| Aigues Mort, | 120 | " | 160 | " | " | ---------- 1 in 1.33 |

Thus, in 1035 cases, we have 592 deaths, or 1 in 1.76. In the Southern States, during the war, where the disease prevailed extensively, one instance is mentioned, where there were 66 deaths in 154 cases; another, where, out of 40, 24 died; and in 35 cases, reported by Dr. S. C. Young, of Grenada, Miss., there was not one recovery. In the Southern States the mortality has been estimated at from 60 to 80 per cent. [Gaillard, *Rich. Med. Jour.*, 1866.] Mons. Gassaud, at Avignon, reports only 2 cases lost in 126! This he attributes to the free and early use of quinia.

*12. Prognosis.*—At *all* periods unfavorable, but especially so during the first four or six days. After this time, the prognosis would seem more hopeful. It is generally more grave in the sporadic than in the epidemic form; and, on the whole, is less grave than in well-marked cases of tubercular meningitis. The question of *diagnosis* will receive consideration in another part of this report.

*13. Complications.*—They are *general* and *special.* Among the former may be mentioned erysipelas, malarial influence, etc. Hirsh and Rummel did not notice any other epidemic or important general disease to coincide with the Dantzig epidemic. Hirsh saw one case complicated with typhus, and another with diphtheria. Allen has seen erysipelas as a common complication. The same observation has been made in various parts of this State during the late epidemic. Dr. Rooker, of Indiana, has seen it occur in connection with diphtheria, and, moreover, has noticed where hog cholera has prevailed most, there this disease has prevailed most.

While many observers, and especially late German observers, have seen the disease rarely complicated with typhus, and deem it a distinct disease from typhus; on the other hand, it has been so constantly associated with typhus in the French epidemics, as to have led many of the French observers to identify it with typhus. This opinion is held by many observers and writers, both in Europe and this country. The examination of the question, as to whether this disease is or is not a form of typhus, will be made in a subsequent part of this report. Such are the most interesting of the general complications which have been observed.

Among the *special* complications, may be mentioned pneumonia, which is the most frequent, perhaps. This complication seems to have been exceedingly common in the epidemic which visited New England in the early part of the present century. Cases of this kind have been often noted. But there does not seem to be any good reason for regarding the coincidence of these diseases as anything more than accidental, as may appear more fully hereafter. Out of nearly 1900 cases, HIRSH has only mentioned *one*. Inflammations of the joints, or of various other parts of the body, and, in some epidemics, of the serous membranes, have been reported by numerous observers. The latter seem to have been strikingly so in some of the French epidemics, and in some of the localities in the New England epidemics in the beginning of this century.

Such are the most important facts of the symptomatical history of "cerebro-spinal meningitis."

### II. POST MORTEM HISTORY.

Here I have been able to collect the evidence afforded by not less than 200 cadavers, which is regarded as affording a sufficient basis for trustworthy generalizations. When they have been given singly and in detail, I have analyzed individual cases. As was observed regarding the symptoms which refer to the nervous system, it may be said of the *post mortem* changes, they are almost never absent from the cerebro-spinal cavity. To this part of the organism, so pointedly referred to by the symptoms, our attention will now be directed, to try if we can find in the after-death appearances any adequate explanation of the morbid phenomena during life.

1. *Central Nervous System and its Envelopes.*—The scalp has been frequently found congested, and beneath it, in some instances, extravasated blood; in other cases, nothing of the kind has been seen. The cranial bones, and especially the diploë, has been found deeply colored with blood in some cases, and, in cases of some duration, NIEMEYER has seen a blue color of the cranial bones, in one case very intense. Occasionally spots of extravasated blood have been found between the cranial bones and dura mater. It has been more common to find effused blood and congestion on the outer surface of the dura mater of the cord, and in the areolar tissue and parts adjacent to it. Such does not occur in a majority of cases, in any marked degree.

1st. *Dura Mater and its Sinuses.*—While I have seen not more than two cases reported, in which the dura mater gave undoubted evidence of structural disease, I have seen no case reported, in which there was not abnormal fulness of its vessels, generally the sinuses and larger veins. In most cases, the sinuses, in particular, have been tumidly filled with blood of a dark color, and, most times, fluid in consistence, or devoid, for most part, of coagula, though not always the latter. *E.G.*, Prof. MAISCH relates a case, in which the blood in the sinuses presented firm coagula, which was true also for the blood in *all* the blood cavities. In *no* case, so far as I have, has seen an exudation been reported on the free or serous surface of the dura mater worthy of note, unless we mention an increase of the serous fluid in the cavity of the arachnoid which is sometimes colored or presents small flocks of lymph, but is usually not changed apparently, except in quantity. This is true for both the cranial and spinal dura mater. They almost never present *well-marked* evidences of *structural change*, or a *firm exudation* on the free or serous surface. Those cases where the congestion and injection of the dura mater was most decided, were found by HIRSH to be the cases where there was the smallest amount of effusion into the ventricles, and *vice versa.* NIEMEYER has seen a case, in which the texture of the dura mater was infiltrated with pus. With these exceptions, and a very few others of like kind, it has uniformly been reported healthy.

2d. *Arachnoid.*—The arachnoid has not often presented evidence of disease. *Generally* about normal, though sometimes discolored, or even apparently thickened and rendered in various degrees opaque. Its freedom from disease is, probably, partly owing to the fact that it is so sparsely supplied with bloodvessels and nerves. The remark just made about its *post mortem* condition, will apply only to the *visceral* layer. The *parietal* layer, or that which lines the dura mater (if we regard this as an actual continuation of the arachnoid,) is almost uniformly healthy in its appearance. The cavity of the arachnoid, many times, does not seem to contain more than its normal quantity of fluid. But most frequently, perhaps, it is increased and sometimes discolored. Occasionally, it is excessive in quantity. When the arachnoid has been roughened, it has generally been the visceral surface of the visceral layer. As regards exceptional cases, NIEMEYER reports one in which the arachnoid was highly injected. He has found the fluid in the cavity of the arachnoid, in a proportion inverse to that in the ventricles. RUMMEL and a few others, have seen the arachnoid adherent to the dura mater; and Dr. G. A. MOSES, of Mobile, is represented in the *Richmond Medical Journal* of this year, as describing the arachnoid as almost always giving evidence of acute inflammation. I have not found it so in my examinations.

3d. *Pia Mater.*—Unlike the two cerebro-spinal investments just mentioned, the pia mater has almost uniformly presented the appearances of disease. Rarely, cases have occurred, in which no evidences of structural change or exudation have been observed, but *not one* where *congestion* has been absent. The rule is, that it presents the most indubitable evidences of disease. Those parts of it most frequently diseased are, at the base of the brain, about the optic commissures; the interpeduncular space behind the commissure; the pons Varolii and medulla oblongata; under surface of the cerebellum; and along the fissures of Sylvius. After this, the surface of the hemispheres is the most constant seat of disease. The congestion and the exudation, when present, may occur uniformly over the surface, or, as invariably happens with the latter, is found along the course of the sinuses, and bloodvessels, and large fissures, and along the fissures between the convolutions. Next, the pia mater of the cervical portion of the cord is most frequently diseased, and of this part and the medulla, the *posterior aspect* is most often diseased. M. LEVY found in 44 cases, the posterior part almost exclusively affected in 27 cases. On the other hand, I have not noticed a case where the anterior half alone was affected. Next in order, the lumbar and lower part of the dorsal regions are most often affected; and, lastly, the pia mater of the dorsal portion seems most frequently to be free from disease. Sometimes cases have occurred, in which the spinal element has been absent, but the converse has been exceedingly rare.

4th. *Exudation—Serous.*—Except in some cases, where death has occurred either with extreme suddenness, or in cases of long standing, there is found to be an increase of the subarachnoid fluid, especially at the base of the brain. In recent cases, it consists in a simple increase of the natural fluid, but in most cases, it is colored yellowish or reddish by pus or blood, and varies in quantity, from a few drachms to several ounces. It varies in consistence and has been found by M. FORGET almost gelatinous. Small flocks of lymph, pus, and blood corpuscles, etc., as already intimated, are often found. It is most abundant where there is the least effusion in the ventricles, and *vice versa.* In cases of several weeks' duration, the membranes have been found unusually dry.

5th. *Exudation—Plastic.*—Except in those cases where death seems to have taken place too soon to permit of such a result, there has been found more or less plastic exudation, most abundant in such places as have been already indicated. It varies in consistence, extent, color, etc. It may be a soft, friable, pus-like layer, or have a gelatinous consistence, or be firmer yet, as coagulated fibrine. It may be nearly transparent, or have a grayish color, being in various degrees opaque, or may have a white or creamy appearance, or be tinged various hues by either pus or blood or both. It may cover a large extent of surface, or be disseminated in patches here and there. But it is always, when present, found along the course of the large vessels and the fissures, and about the roots of the nerves, whether the cranial or spinal, the posterior roots of the latter, in particular. It is, when present, on the surface of the pia mater, but may be infiltrated into its structure, or beneath it, into the nervous substance for a short distance. It varies in thickness, from a mere film to a line or two, or more. Its consistence depends much on the duration of the case, being most so in cases which have some duration.

In the exudation the microscope has revealed free nuclei, and thin walled cells containing two or three nuclei. [MAISCH.] In the *American Medical Times*, for April 30th, 1864, a case is reported by Dr. FROTHINGHAM, in which the exudation showed no pus corpuscles. But WUSCHENDORFF and other observers have seen pus corpuscles, as we will see hereafter, perhaps.

6th. *Ventricles.*—The ventricles have generally been observed with more than the normal quantity of fluid in them; occasionally, the quantity has seemed to be normal. It has been found to vary from a small increase to several ounces, and in a case reported by Dr. READING, the quantity was so great the ventricles were ruptured. M. TOURDES saw, in 44 cases examined at Strasbourg, 26 in which the ventricles were distended; and in 44 cases examined by M. LEVY, at Val de Grace, 18 presented distended ventricles. NIEMEYER mentions a case, where the ventricles contained as much as six ounces of fluid.

The character of the exudation has varied from clear serum, sometimes mingled with pus, or colored with blood and the like, [FORGET, LEVY, BROUSSAIS, HIRSH, RUMMEL, NIEMEYER, DRAPER, etc.,] to the purulent and gelatinous exudation already described as occurring on the pia mater, and which frequently extends into the anterior and posterior, and even the middle, horns of the lateral ventricles, and is found in the other ventricular cavities. [NIEMEYER.] The exudation generally occupies the *floor* of the ventricular cavity.

The walls of the ventricles are frequently injected, which seems most often true of the roof of this cavity. The plexus choroides is nearly always injected and bathed in the exudation, as the optic thalami and striated bodies also are. The ventricular walls have been found softened, and ALLEN speaks of having seen ecchymosed spots in them.

7th. *Nervous Substance.*—This, in the majority of cases, has been reported as healthy. Occasionally it has been seen paler than natural, in cases of some standing in particular. [NIEMEYER and others.] More frequently it has been seen congested in various parts and degrees. FORGET has found in chronic cases, a turgescent state of the brain matter, and in more recent cases, some injection of the nerve substance. The same appearances are, substantially, given by M. BROUSSAIS. M. LEVY found in 44 cases, injection of the nervous substance 9 times. NIEMEYER has met with cases, in which the brain was of a dark or a light red color. HIRSH has seen a case, in which the brain generally was injected; one in which the great ganglia, at its base, and another, in which the cerebellum was alone injected. LIDDELL reports congestion in one case; and the cases reported by Dr. READING were all of them more or less so. The latter observer twice saw what seemed to be an abscess of the cerebellum—collections of pus in the cerebellum—in one case, nearly a gill. The *spinal cord* sometimes participates in this congestion.

*Softening*, has occasionally been reported in chronic cases. NIEMEYER records it in 2 cases out of 15. One of the cases was of 49 days standing, and exhibited cheesy metamorphosis. M. CHAUFFARD observed softening, comparatively often, at Avignon. DRAPER mentions one case of partial softening. In three of Dr. READING'S cases, the brain was softer than natural. The brain and spinal cord have rarely been seen œdematous. NIEMEYER and DRAPER, each report a case. Dr. HUTCHINSON reports a case, in the *American Medical Journal*, for July, 1866, etc. The convolutions have been observed flattened in long-standing cases, by M. FORGET; also, he has seen the convolutions adherent, one to another. A similar case is reported by NIEMEYER. The same remarks are true, though less frequently, of the spinal cord.

2. *The Chest.*—The chest has yielded no characteristic evidences of disease, and, in the majority of cases, perhaps, nothing strictly abnormal. In many cases, the common hypostatic congestion has been observed in the lungs. M. LEVY has seen 9 cases out of 44. But occasionally the lungs have been found deeply congested with dark blood. Such was true in the case reported by Dr. LEVICK, and in two cases reported by Dr. LIDDELL. The latter gentleman found about four ounces of serum in either pleural cavity in one case. Pneumonia was found twice by LEVY, in 44 cases. Dr. READING reports 1 case in 7, where marks of pleuro-pneumonia were present. In 3 of the 4 cases examined by Dr. J. C. WARREN, he found extensive thoracic lesions; in one, double pleurisy, with extensive purulent deposits. M. BOUDIN says, it was no uncommon thing to have purulent deposits in any or all the serous cavities, especially the tunica vaginalis. The pericardium has been occasionally found congested, as in one case reported by GERHARD, and one each by READING and WARREN. M. BILLERY, at Grenoble, speaks particularly of the lesions of the pleural and synovial cavities. In the case of GERHARD and some few others, spots of extravasated blood, were seen in the structure of the pericardium. Others have found the amount of serum increased, even to several ounces. [LEVY, FROTHINGHAM.] This comprises most of the thoracic lesions I have noticed. They were far more common in the French and New England epidemics than I have learned them to have been elsewhere. In the late German epidemics, *e.g.*, they were comparatively unknown.

3. *Abdomen.*—The organs within the abdomen have usually presented nothing strikingly abnormal. The stomach has almost uniformly been found healthy or paler than natural.

NIEMEYER has seen the mucous coat congested. In the majority of cases the small intestines give some evidence of disease. This is generally slight hyperæmia, especially in the neighborhood of vascular parts, as the patches of Peyer and the intestinal glands. LEVY reports slight enlargement of Brunner's and Peyer's glands in about one-third of the cases he saw. Much the same, by FORGET and BROUSSAIS. The former saw one case of ulceration of the intestines. NIEMEYER reports much the same. TOURDES, at Strasbourg, saw two cases of ulceration in 46, and 32 of the cases showed some slight follicular lesions, while 8 showed none of any kind.

Hemorrhagic or ecchymosed spots have been found occasionally. Once by NIEMEYER, in the great end of the stomach. In a few cases, ecchymosed spots have been observed in the intestinal walls, or mesentery, etc. [LEVICK, JEWELL, (of Pa.) NIEMEYER.]

The liver, spleen, and kidneys have generally been found healthy. But cases of congestion in various degrees are reported. [NIEMEYER, RUMMEL, LIDDELL, etc.] In Dr. LEVICK'S case, the kidneys and bladder presented ecchymosed spots. In two of Dr. LIDDELL'S cases, the bladder contained urine charged with albumen. The urine, under such circumstances, was observed by NIEMEYER to throw down a copious sediment In 4 cases, in which the kidneys were submitted to the microscope, Dr. DRAPER reports fatty degeneration. It was observed in one case, that of a little girl who died 44 hours from date of attack. There were no signs of previous disease in these cases. The serous membrane lining the cavity of the abdomen, has generally been found healthy. In some cases, it has presented evidence of disease. This was true, for example, in some of the epidemics in France.

4. *Vascular System.*—The heart and bloodvessels have seldom presented anything worthy of note. The heart has sometimes been found paler than natural, in others increased in color, and, rarely, softened. In the large majority of cases, the blood found in the cavities of the heart and bloodvessels has been darker and more fluid than natural. In a few cases, extreme fluidity has been observed. [JEWELL, (of Pa.) LEVICK, LIDDELL.] But M. FAURE VILLAURE, at Versailles, found generally large yellow fibrinous clots, in the heart in particular. The same was often observed at Strasbourg, in the province of Landes, Nancy, Lyons, Colmar, etc., in 1837. The blood drawn during life at these places, exhibited much buffy coat. In the case reported by GERHARD, small coagula were observed, as they also were in the cases reported by Dr. DRAPER. NIEMEYER has seen clots in several cases; and in one case examined by Prof. MAISCH, already referred to, *very firm clots* were found throughout the vascular system, *even in the sinuses* and large vessels within the cerebro-spinal cavity.

Various other appearances have been noted by observers. The muscles have most times, perhaps, appeared natural, occasionally paler than natural. A few observers report them redder than natural. [GALLUP.] The muscles which have been contracted, as those of the back of the neck, have been found redder, or darker, and softer than natural. The same muscles were observed by RUMMEL to be least rigid after death. Extravasated blood has been observed, not unfrequently, in spots in the structure of various organs.

Such are the *most important post mortem* appearances noticed by observers, either in this country or Europe. This summary of facts, as remarked in the beginning, has been drawn from not less than 200 cases.

III. ETIOLOGICAL AND PATHOLOGICAL HISTORY.

Proceeding as we only can safely, as a rule, in medicine, from *effects* to *causes*, with the materials and facts from the foregoing analysis before us, we are ready to begin the discussion, as to the *causes* and *nature* of the disease. In doing this, I shall invert the order of discussion observed thus far, in beginning with certain general propositions, and under each shall collect the particular evidence on which it rests. These propositions are really the conclusions reached, but have been arranged in the manner which follows, for convenience of discussion. The nature of a disease is to be determined from the phenomena or *effects* perceived and its *causes*. The former I have endeavored to show, in part, in the above analysis. The *effects* having been presented, let us now turn to the *causes*, as the next step in the enquiry.

In the class of diseases to which this seems to belong, to determine causes, satisfactorily, is confessedly one of the most difficult tasks in pathology, and nowhere is more *positive* light required, than on the question of epidemic causes in general. Such causes, while they are clearly matters of inference, are, thus far, mainly *unknown*, as regards positive evidence. We are obliged to admit their existence, but can offer but little, if any, direct proof of it.

1. I proceed, then, to remark, in the first place:—That the disease depends primarily, in all probability, on a specific external epidemic cause, unknown, save in its effects. Various known external causes, either singly or combined, have been assigned to this disease, such as cold, foul air, social misery, insufficient or bad food, change from civil to military life, fatigue, nostalgia, abuse of alcoholics, depressing agencies of various kinds, heat of the spring sun, crowding in small houses with defective ventilation, and many others, have been assigned by different observers, not only as *predisposing*, but also as *efficient* or *essential* causes. That such agencies may and do predispose the system to the inroads of disease, whether the one under consideration or any other, by impairing the energies of the organism, there can be no reasonable doubt. But that any of the causes of disease mentioned above, or others of a like kind, are capable of *originating* this disease, seems exceedingly doubtful, as will appear from the following considerations:—

In general, it may be remarked, in relation to such assumed causes, that they exist thousands of times without either producing or being accompanied by "cerebro-spinal meningitis." This is a matter of almost daily observation, and to suppose they should at one time produce the disease, and *not* at other times, is destructive of the very idea of causation. On the other hand, the disease has prevailed many times in the absence of, at least most, if not all these assumed causes.

The disease, for example, as regards cold, has often been prolonged into the hot season, and has even begun as an epidemic, and prevailed extensively, in midsummer, as many as eight times, in France. It has prevailed in rural districts as well as civic districts, and has even seemed to prefer rural districts in some cases, as in our own late epidemic. It has appeared among both rich and poor; on the uplands as well as lowlands; among those well fed, as well as among those poorly fed; among those who have enjoyed thorough ventilation, as well as those under an opposite condition; among the civil, as well as military population, and so in relation to the whole catalogue of these known assumed causes, of which the malady seems to be wholly independent, except that they may, in a general way, predispose to, or intensify it. As we shall see, the disease has an individuality sufficiently well marked and constant to necessitate the conclusion, in so far as it presents constant phenomena, that it is due to a constant cause, which cannot be alleged of any known cause. For *not one* of them has been observed to be constantly associated with the disease. Since, then, we are bound to admit a cause, we can do no other than conclude, if the essential cause be external or exopathic, it is one, which, though unknown directly, is special, and, by reason of its apparent independence of known external conditions, and its wide field of operation, must be assigned to that obscure but admitted class, *epidemic causes.*

But the cause may not be external. It may be internal or autopathic. Such causes may be either *predisposing* or *essential.* Our concern at this time is particularly with the latter. Among autopathic causes, may be mentioned:—

1st. *Age.*—While no age has been exempt, the young have been the principal victims, and, as a general thing, the younger the age, the greater the mortality. The period between 10 weeks and 7 years, has furnished a greater mortality than any later period of equal length. But, in Berlin, according to HIRSH, the disease was mostly confined to the adult population. In other instances, the period from 18 to 24 has yielded a larger mortality, than from 10 to 18. But, as a whole, the disease has prevailed vastly more among the young, vigorous, healthy, and athletic, than among the aged or infirm. At Belfast, *e.g.*, Dr. MAYNE says, the disease was almost exclusively confined to boys, between the ages of 7 and 12. Most of the cases seen by HIRSH, were below 15. The same was observed in the epidemics in Denmark and Sweden, and at Stettin and Bromberg. Reports of the same tenor, have been made by NIEMEYER, RUMMEL, and others. At Rochefort, according to LEFEVRE, the greatest mortality was between the ages of 30 and 40 years; at Strasbourg, between 14 and 21; at Aigues

Morte, Val de Grace, Gibraltar, Lisle, etc., much the same. For further matter on this topic, see the paragraph on mortality, above. It must be remembered, however, that the mortality reports made in France, in some cases refer almost exclusively to the military population, in which case we would, of course, expect the greatest mortality at a period necessarily different from what we might have it among the civil population.

2d. *Sex.*—All reports, so far as I have seen, represent the disease as prevailing most among males—in a few cases, almost exclusively so.

3d. *Hereditary Influence.*—There are no good reasons to suppose they play any part as essential causes of the disease, though it is possible they may exert some influence as predisposing causes.

But, in this case, it may be, something has been *retained* in the blood, which should normally have been removed from it by some one or more of the emunctories of the body, as urea, *e.g.*, by means of which the blood is poisoned, and becomes the cause of the disease. Or, the blood *may* have undergone some change in *quantity*, either as a whole, or in respect of some one or more of its constituents. Or, some change may have taken place in the *quality* of the blood or some of its constituents, in which cases it might be rendered unfit, on the one hand, to maintain a healthy state of the structures, and, on the other, a positive cause of disease.

As regards the first case, it may be remarked, that so far as the evidence goes, there does not appear to be any change in the secretions, either as a whole or in respect to their ingredients, sufficient to account for the phenomena of this disease. The urine, for example, has usually been reported healthy in appearance, and nothing concerning the other secretions worthy of note.

It is true a few observers have seen, especially in the later stages of the disease and during convalescence, albumen in the urine, and sometimes a deposit, more or less copious, of either the urates or the phosphates. [NIEMEYER, ROOKER.] But other observations do not accord with these, and, besides, the same conditions appear in forms of disease not even analogous to this. It must also be remembered, that it generally requires some time to effect such a change in the secretions as, in this way, to produce marked symptoms, while this disease frequently has its whole progress from a state of apparent health to a fatal termination in a few hours. And it would be of no avail in the present case, were we to show that even marked alterations of the secretions had occurred *during* the *course* of the disease, unless it could also be shown they appeared before the onset of the disease. The evidence is pretty conclusive that the essential cause of the disease is not to be found in retained secretions.

2. That the blood has been deprived to any considerable extent of its elements, or that there has been a considerable quantitative change at any stage of the disease, does not appear from the evidence to be a fact, or even probable in any noteworthy degree.

3. That some change has been wrought in the blood or some of its chief constituents, by which it acts unhealthily on the living structures of the body, seems highly probable, as will appear from the following facts and considerations. The particular changes which have been observed under different circumstances and at different times, are as follows:—

There has been found in all the cases examined, uniformly, an increase of fibrine, and, generally, of the corpuscles, as we have already seen, before death. As regards the condition of the blood after death, we have seen that, while in the large majority of cases the blood is not only darker, but also more fluid than usual, that, on the contrary, in some well-marked cases, it has not been so, as in the case of Prof. MAISCH, in which the blood was firmly coagulated in the heart and vessels. But that this change of color and fluidity noticed in the blood of most cases after death, is not a *cause* of the disease, but, on the contrary, only a *part* of the disease, seems quite certain, for the evidence goes to show a *contrary* state of the blood during the life of the patient.

As already remarked, the corpuscles are notably increased, as shown in the cases examined, from 131, about the normal standard, to 136 or even 142 parts in 1000. But whether or not they have undergone any change *per se* is matter of conjecture, since the evidence does not show. But, in most cases, it would seem hardly possible for a change to occur, either here or in the fibrine, or any other known constituent of the blood, with the suddenness which would be required to explain the frequently abrupt onset of the characteristic symptoms of the disease. Except the *color* of the blood before death, which is unusually bright, generally, and a very few unimportant facts relating to other changes, neither striking nor constant, these are all. Now, so far as we know, blood-changes quite similar to these take place in other forms of disease different from this, and in whatsoever relation we regard these blood-changes to the disease, they must have a cause, and this cause can scarcely be other than external. But we have found already that there is no evidence that the ordinary or positively known causes of disease have any direct share in its production.

It is thus seen, that the probabilities are against the existence of any *positively known* external cause, and that what is known of the state of the blood and secretions would hardly justify us either to say that the blood, because of its changes, became the *cause* of the train of symptoms and morbid appearances observed, or were *themselves initiated* by an internal cause. If these remarks be just, it must follow that we adopt, as the only alternative, an external cause as the essential one. That this must be the truth, farther appears, because:—

1st. The disease is so widespread in its prevalence, and occurs under such a variety of circumstances, and in these respects so nearly resembles undoubted epidemic diseases, as cholera, and presents such characteristic phenomena as to make it alike impossible for us to explain them by any known external cause or by any changes in the blood, the which, if they do occur, constitute rather a *part* of the disease than the initial or starting point in causation. We are thus forced to hypothecate a cause, such as the phenomena require.

2d. The suddenness of the attack is *so* marked, and its progress to a fatal termination so rapid, and the affection of the ner vous system, as shown by the symptoms and the *post mortem* appearances so early and so constant, as to beget the belief that some energetic poison has been introduced into the system, because we know how many poisons may be and have been taken into the system, which, if they have not produced the same, have, nevertheless, produced symptoms so far similar as to leave no reasonable doubt in our minds that the symptoms of this disease have been produced in some similar way.

The leading symptoms and appearances are so constant, the sphere of operation so wide, and the attack, in many cases, so sudden, as to require a generality and uniformity of causation, which can be secured in no other way than by admitting as true the proposition we began with. This examination of the question of causation will not appear superfluous to those who are familiar with the discordant opinions held on this subject.

The next proposition I would lay down, is:—That it is a disease *sui generis*, a disease essentially different from and independent of other recognized forms of disease.

That this is not the opinion generally held, will appear by showing what are the opinions which have been and still are held by numerous eminent members of the profession, both in this country and Europe. That the disease is only a form of typhus, has been held by a large majority of European writers, perhaps, as well as by many in our own country.

Among the former, may be mentioned HILDENBRAND, PRINGLE, OZANAM, SYDENHAM, LEFEVRE, MATTOT, BILLERY, CHAUFFARD, BOUDIN, BROUSSAIS, MURCHISON, AITKIN, and many others. Among the latter, WOOD, DRAKE, DRAPER, and others.

That the disease is simply a form of the ordinary "congestive" or "pernicious" malarial fever, has been the opinion of many physicians practising in the paludal districts of the West, and of some few *writers*, apparently, as MARSHALL HALL, (Prac. Med.) Others have supposed the disease identical with erysipelas in its essential nature. Others have supposed the disease simply malignant scarlatina; while another thinks it quite probable the causes of diphtheria and hog-cholera are identical with the cause of this disease. On the other hand, several observers in this country and Europe believe it to be a disease *sui generis.*

These diversities of opinion show us the necessity of a careful examination of the *facts* relating to the disease. It is for want of this that we have such contrariety of opinion. While a broad survey and analysis of the facts from different times and places will show that a *certain group* of symptoms and appearances are never absent in any case, it will, on the other hand, show that the disease has often had imparted to it a peculiar hue or tendency, so to speak, by the prevailing "constitutions" and diseases, as the typhus, malarial, etc., so much so, many times, as almost to mask the characteristic symptoms of the disease, as was observed in relation to typhus in the French epidemics. Or, as we have seen, it may be complicated with some other form of disease, as pneumonia, as was observed in the epidemic which prevailed in New England, in the early part of the present century. The only one of the opinions above referred to which seems to demand a careful examination, is that which holds "cerebro-spinal meningitis" to be identical with typhus. That the two diseases are not identical, will appear by drawing a parallel between their symptomatical, *post mortem*, and etiological histories:—

| CEREBRO-SPINAL MENINGITIS. | TYPHUS FEVER. |
|---|---|
| I. *Symptomatical History.* | *Symptomatical History.* |
| 1. The attack is sudden and the progress, as a general thing, rapid. A few exceptions occur. | 1. Attack almost never sudden. Progress generally slow. Average duration 21 days. The "blasting typhus," which visited the French army at Saragossa, Torgau, and Wilna, were simply unusual *exceptions*, and as such they do not establish a *rule.* |
| 2. The secretions not much disturbed, as a general thing. | 2. Secretions almost always much disturbed. |
| 3. Generally introduced by a decided or severe chill. | 3. Generally begins with rigors, but not often decided chill. |

| | |
|---|---|
| 4. Heat of surface does not, on the average, rise above normal. Color, usually not far from natural. Face generally pale in early part of disease, but sometimes flushed or cyanotic. | 4. Heat of surface *does* rise above natural, the average being so high as 107° Fah. "Calor mordax," surface and especially *face*, always *dusky*, often positively muddy. Face usually flushed, and expression dull. |
| 5. Spots do not often appear. Vary as to kind, duration, time of appearance, disappearance, etc. Seldom elevated. No sudamina. | 5. Spots pretty constant and generally elevated (rubeoloid.) Appear from 5th to 8th day. Remain for 11 or 12 days. Above 20 years, spots all but constant. |
| 6. The blood drawn during life has always shown a firm coagulum, bright color, and often a decided buffy coat. Analyses have shown a marked increase of fibrine and corpuscles. | 6. Blood drawn during life loosely coagulated. No buffy coat worthy of note. Fibrine and corpuscles both evidently deteriorated. The *former* diminished and the corpuscles variable. |
| 7. Tongue generally not brown or dry, unless in later stages. | 7. Tongue generally brown or dry almost from commencement. |
| 8. Vomiting almost a constant symptom early in disease, almost exclusively sympathetic. Bowels generally normal or constipated. | 8. Seldom vomiting. Diarrhœa frequently. |
| 9. Respiration not always, but on the average, *slower* than natural. | 9. Respiration not always, but on the average, more frequent than natural. |
| 10. Urine generally not far from healthy in quantity and quality. | 10. Urine seldom normal in quantity or quality. |
| 11. Tonic spasm of extensors of back of neck, and back, and flexors of limbs, almost al- | 11. Neither tonic or clonic spasms are present, only very rarely. |

| | |
|---|---|
| ways present. Clonic spasms not unfrequent. Paralysis of motion sometimes present. | |
| 12. Hyperæsthesia is quite frequent and often general. Anæsthesia sometimes present. | 12. Marked hyperæsthesia seldom present. The same of anæsthesia. |
| 13. Pain in the head often "*terrible*," and always *severe.* Worst in *back* of head, neck, and spine. | 13. Pain in the head *not* severe usually, and more often in *front* of the head. |
| 14. Special senses *often* markedly disturbed, and in many ways. | 14. Special senses *not* markedly disturbed. |
| 15. Delirium almost constant in early part of the disease, and *active.* Mental faculties and senses not always dull. | 15. Delirium *not* constant and not always active, but low or muttering, rather. Mental faculties and senses almost *always* dull. |
| II. *Post Mortem History.* | *Post Mortem History.* |
| 1. The only constantly diseased appearances are those found in the cerebro-spinal cavity. | 1. No constant diseased appearances, unless those of the blood. "*Other* lesions may be considered incidental." [WOOD. |
| 2. Pia mater constantly shows unhealthy appearances. | 2. Does not show such often. |
| 3. Exudation always present where time is given. | 3. Exudation *not* always present; on the contrary, comparatively seldom. |
| 4. Ventricles often diseased. | 4. Ventricles seldom diseased. |
| 5. Serous exudation frequently abundant. | 5. Seldom abundant. |
| 6. Blood generally dark in color and fluid in consistence, but not always. | 6. Blood *always* fluid and dark. |
| III. *Etiological History.* | *Etiological History.* |
| 1. Is *not essentially* affected | 1. *Is* affected essentially by |

| | |
|---|---|
| *by*, or dependent *on*, *known* external conditions. | known causes and external conditions. |
| 2. Depends on a peculiar epidemic *external* cause, without reasonable doubt. | 2. Does *not* seem to depend essentially on a peculiar external epidemic cause. |
| 3. Affects all ages and both sexes, but prevails *most* among males, and the *young*, vigorous, and healthy. | 3. All ages and both sexes, but *most* among *adults*. |
| 4. Prevails in all conditions and districts—in the country, as well as the city. | 4. Almost exclusively among the poor, ill-fed, and in over-crowded localities; comparatively seldom in the country. |
| 5. Neither contagious nor infectious, in all probability. | 5. Certainly contagious under some circumstances, and is probably infectious. |

[*Continued in the next No.*]

